# Development and Validation of a Nomogram for Estimating Stroke Risk in Nonvalvular Atrial Fibrillation Patients With Left Atrial Stiffness: A Single‐Center Cross‐Sectional Study

**DOI:** 10.1155/cdr/8877050

**Published:** 2026-07-16

**Authors:** Decai Zeng, Xiangling Cao, Yongzhi Cai, Tongtong Huang, Shuai Chang, Bingling Wu, Ji Wu

**Affiliations:** ^1^ Department of Ultrasonic Medicine, First Affiliated Hospital of Guangxi Medical University, Nanning, China, gxmu.edu.cn; ^2^ Department of Rehabilitation Medicine, First Affiliated Hospital of Guangxi Medical University, Nanning, China, gxmu.edu.cn

**Keywords:** left atrial stiffness, nomogram, nonvalvular atrial fibrillation, stroke

## Abstract

**Background:**

Nonvalvular atrial fibrillation (NVAF) is a prevalent arrhythmia associated with an increased risk of ischemic stroke. This study is aimed at assessing the clinical significance of echocardiographic left atrial (LA) stiffness and developing a nomogram integrating clinical and echocardiographic parameters to evaluate individualized stroke risk in NVAF patients.

**Methods:**

We enrolled 402 consecutive NVAF patients between January 2020 and March 2023. Clinical and echocardiographic data were collected, and patients were randomly assigned to training (*n* = 281) and validation (*n* = 121) datasets. Univariate and multivariate logistic regression identified independent stroke risk factors, which were used to construct a nomogram. Model performance was evaluated by receiver operating characteristic (ROC) curve, calibration curve, and decision curve analysis. The reclassification ability of the nomogram was assessed using integrated discrimination improvement (IDI) and net reclassification improvement (NRI).

**Results:**

Logistic regression identified LA stiffness, LA reservoir strain, hypertension, peripheral vascular disease, and anticoagulation as independent factors associated with stroke. The nomogram based on these factors showed excellent discrimination, with a C‐statistic of 0.889 (0.847–0.924) in the training cohort and 0.880 (0.808–0.932) in the validation cohort. Model A (LA stiffness, LA reservoir strain, hypertension, peripheral vascular disease, and anticoagulation) provided better net reclassification and integrated discrimination than Model B (LA stiffness, LA reservoir strain, hypertension, and peripheral vascular disease) despite both outperforming the CHA_2_DS_2_‐VASc model. LA stiffness was inversely correlated with left atrial appendage (LAA) emptying (R = −0.52, *p* < 0.01) and filling velocities (R = −0.50, *p* < 0.01). Patients with LA thrombus or spontaneous echocardiographic contrast exhibited significantly higher LA stiffness (*p* < 0.05).

**Conclusion:**

A nomogram incorporating LA stiffness, LASr, hypertension, and peripheral vascular disease can effectively identify the risk of stroke in NVAF patients. Additionally, a secondary model including anticoagulation showed further improved performance but requires prospective validation. Elevated LA stiffness might play a role in the development of stroke in NVAF by influencing LA appendage hemodynamics.

## 1. Introduction

Atrial fibrillation (AF) is a major risk factor for stroke, primarily due to the formation of thrombi within the left atrial appendage (LAA), which can subsequently embolize to the cerebral circulation [1]. The strong association between AF and stroke is well established, and echocardiography plays a central role in assessing the cardiac structures and functions that contribute to this risk [2].

The CHA_2_DS_2_‐VASc score remains the cornerstone for stratifying stroke risk and guiding anticoagulation therapy in patients with AF. However, this scoring system relies exclusively on conventional clinical risk factors and does not incorporate markers of subclinical atrial dysfunction, which can be significant contributors to thromboembolic events [3].

Echocardiographic assessment, especially through transesophageal echocardiography (TEE), plays a vital role in identifying intracardiac thrombi—most notably in the LAA—which directly influences anticoagulation strategies and interventional decisions such as cardioversion [4]. However, since TEE is semi‐invasive and can cause discomfort or complications, there is a significant need for noninvasive alternatives.

In patients with AF, impaired left atrial (LA) function strongly predisposes to thrombus (TH) formation by promoting blood stasis. Transthoracic echocardiography, especially when enhanced by speckle‐tracking technology, provides a noninvasive method to quantitatively assess LA mechanics, including LA strain, and offers valuable insights for thrombosis risk stratification and management [5].

Elevated LA pressure is another key factor in the pathophysiology of AF, facilitating both the initiation and maintenance of the arrhythmia through electro anatomical remodeling [6]. The presence of LA fibrosis compounds this process by altering atrial hemodynamics—most notably, by reducing wall shear stress and increasing the oscillatory shear index—which fosters a prothrombotic environment, as demonstrated by computational fluid dynamic studies [7]. Both LA hypertension and fibrotic remodeling have been independently associated with adverse clinical outcomes, underscoring the importance of a comprehensive assessment that incorporates both functional and structural LA parameters [8].

The noninvasive evaluation of LA pressure and stiffness using advanced echocardiographic techniques, such as speckle‐tracking, offers promising avenues for refining stroke risk assessment in AF patients [9, 10]. Integrating these echocardiographic indicators with clinical risk factors may significantly enhance the predictive accuracy of current risk stratification models.

In recent years, nomograms have become a widely used predictive tool in medical research because they transform the contributions of individual variables into scores, allowing for intuitive calculation of event probabilities. Therefore, the development and validation of a nomogram that combines clinical and echocardiographic parameters—particularly LA stiffness assessed by echocardiography—have the potential to improve the accuracy of stroke risk estimation for patients with nonvalvular atrial fibrillation (NVAF). The present study is aimed at evaluating the clinical utility of LA stiffness measured by echocardiography and developing a comprehensive nomogram that integrates both clinical and echocardiographic data for individualized stroke risk assessment in patients with NVAF.

## 2. Methods

### 2.1. Study Subjects

This cross‐sectional study consecutively enrolled patients with NVAF who underwent AF catheter ablation at the First Affiliated Hospital of Guangxi Medical University between January 2020 and March 2023. Inclusion criteria included patients aged over 18 years with paroxysmal or persistent AF. Exclusion criteria comprise congenital heart disease, cardiomyopathy, moderate to severe valvular heart disease, prior valve repair or replacement, acute coronary syndrome, previous catheter ablation, and stroke caused by other reasons, such as carotid artery stenosis > 50%, unstable carotid artery plaques, and cerebrovascular lesions. Routine transthoracic and TEE were performed.

### 2.2. Definition of Stroke and Grouping

The risk of stroke is defined as having a history of stroke or transient ischemic attack (TIA) at baseline. The diagnosis was confirmed based on medical records and cranial imaging (CT or MRI). All events were adjudicated by two independent cardiologists blinded to echocardiographic data, with disagreement resolved by consensus. All patients were divided into two groups based on the presence of a documented history of stroke/TIA: the stroke group (*n* = 70) and the nonstroke group (*n* = 332). This study received approval from the Ethics Committee of the First Affiliated Hospital of Guangxi Medical University (Approval Number: 2022‐KT‐077), and written informed consent was obtained from all participants involved in this study.

### 2.3. Clinical Baseline Data and Laboratory Tests

General information of the patients was collected, including gender, age, height, weight, blood pressure, medical history (hypertension, coronary heart disease, diabetes, dyslipidemia, peripheral vascular disease, stroke/TIA, etc.), and history of anticoagulant medication use. Anticoagulation is defined as the use of oral warfarin or novel oral anticoagulants. The CHA_2_DS_2_‐VASc score for each patient was calculated; a higher score indicates a greater risk of stroke.

Upon admission, fasting venous blood samples were obtained for the evaluation of biochemical markers, such as N‐terminal pro‐brain natriuretic peptide (NT‐proBNP), troponin I, serum creatinine (SCr), estimated glomerular filtration rate (eGFR), and others. The eGFR was determined using the CKD‐EPI equation, which incorporates SCr, age, sex, and race.

### 2.4. Transthoracic Echocardiography

Patients were positioned in the left lateral decubitus position, and standard 6 cardiac cycles of images were collected with continuous electrocardiogram monitoring. All enrolled patients underwent transthoracic echocardiography using the PHILIPS EPIQ 7C ultrasound diagnostic equipment, with S5‐1 transducer (frequency: 1–5 MHz) for transthoracic echocardiography.

**Figure 1:**
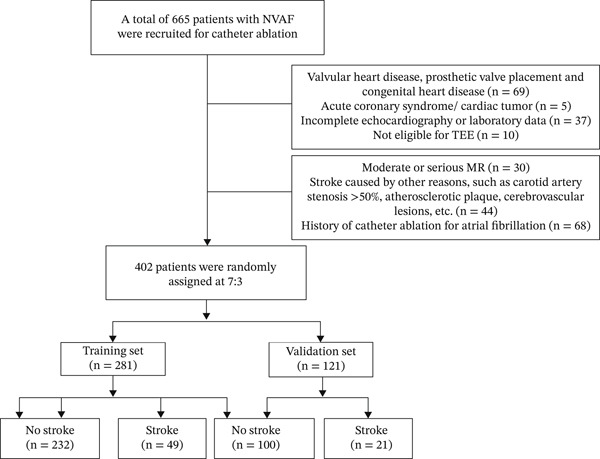
Flowchart of patient selection.

A transthoracic three‐dimensional matrix probe was used to acquire images with the Heart Model (HM) in the standard apical four‐chamber view (as shown in Figure 1). The HM mode was utilized to acquire three‐dimensional volumetric data of the left heart, including left ventricular end‐diastolic volume (LVEDV), left ventricular end‐systolic volume (LVESV), and ejection fraction (EF). The left ventricular (LV) mass was calculated using the following equation: LV mass = 0.8 × {1.04 × [(SWT + LVEDD + PWT)^3^ − (LVEDD)^3^]} + 0.6, where SWT represents septal wall thickness at end‐diastole, LVEDD denotes left ventricular end‐diastolic diameter, and PWT indicates posterior wall thickness at end‐diastole [11]. The left atrial volume index (LAVI) and the left ventricular mass index (LVMI) were determined according to each patient′s body surface area. To assess the peak velocity (E) of early diastolic mitral inflow, the apical four‐chamber view was employed, and the Doppler sample line was placed 1 cm below the mitral valve orifice to measure the early diastolic peak blood flow velocity. Tissue Doppler imaging is then employed to measure the e′ velocity at both the septal and lateral sides of the mitral annulus, and the average e′ value is calculated. Finally, the average E/e′ ratio is determined by dividing E by the average e′ value.

### 2.5. LA Strain Analysis

**Figure 2:**
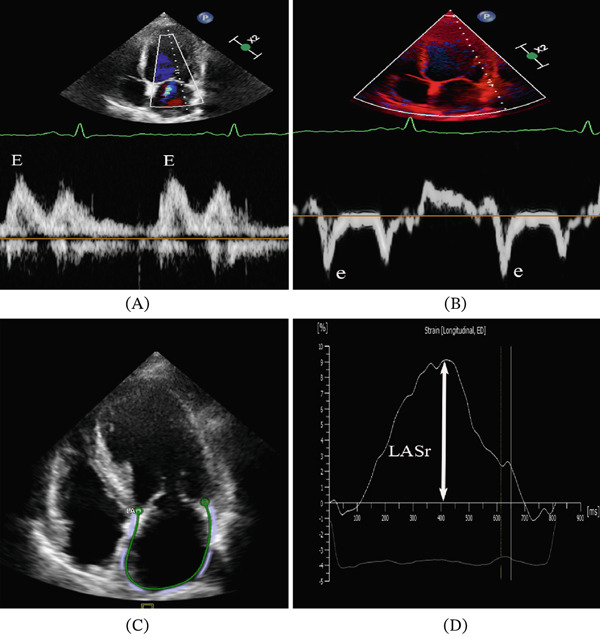
The assessment of left atrial stiffness by echocardiography. (A) Measurement of mitral inflow E‐wave velocity. (B) Evaluation of lateral mitral annular e′ velocity. (C) Analysis of left atrial strain. (D) Left atrial reservoir strain as shown on the left atrial strain curve.

Following guidelines, the onset of the QRS complex was taken as the reference point (R–R gating) for measuring LA strain using two‐dimensional speckle tracking technology. With the apical four‐chamber view, the endocardial borders of the left atrium were traced from the annulus of the mitral valve along the endocardium of the left atrium, excluding the pulmonary veins and LAA, to the other side of the mitral valve annulus. The region of interest (ROI) width was set to 3 mm, adjusting the size and shape of the ROI to include the thickness of the LA wall while excluding the pericardium. The LA strain curve was obtained, and measurements of left atrial reservoir strain (LASr) were made. LA stiffness was calculated as the ratio of E/e′ to LA reservoir strain, LA stiffness = (mean E/e^′^)/LA reservoir strain, as shown in Figure 2.

### 2.6. TEE

The TEE was performed using the X5‐1 transducer (frequency: 1–5 MHz) and was mainly used for precatheter ablation TH screening to rule out surgical contraindications. The patient fasted for more than 8 h prior to the procedure. Topical pharyngeal anesthesia was administered using dyclonine gel. The patient was positioned in the left lateral decubitus position, and the probe was placed at the midesophageal level. The left atrium and LAA were examined from multiple angles (0°, 45°, 90°, and 135°) for the presence of spontaneous echo contrast (SEC) or TH formation. SEC was defined as a swirling, smoke‐like appearance of blood flow in the LA and/or LAA. Pulse Doppler was utilized to measure the peak flow velocity during both the filling and emptying phases of the LAA. The sample line was positioned at the center of the LAA orifice in the 45° plane in midesophageal short axis.

In patients with AF, hemodynamic parameters were measured over five consecutive cardiac cycles to obtain reliable and consistent data. During sinus rhythm, Doppler measurements were averaged over three consecutive cycles. To assess the intraobserver and interobserver reproducibility of mean E/e′ ratio, LASr, and LA stiffness measurements, intraclass correlation coefficients (ICCs) were calculated based on measurements from 30 randomly selected patients. These measurements were performed by two independent observers (to determine interobserver variability) and by the same observer at two different time points (to determine intraobserver variability).

### 2.7. Statistical Analysis

All statistical analyses were performed using R software (Version 4.2.1). For continuous variables with a normal distribution, comparisons were made using the Student′s t‐test, and results are expressed as mean ± standard deviation. Continuous variables that did not follow a normal distribution are presented as median and interquartile range (Q1 and Q3), and were compared using the Mann–Whitney test; these results are reported as median with interquartile range. Categorical variables are described as frequencies and percentages, with group comparisons conducted using either the *χ* ^2^ test or Fisher′s exact test, depending on suitability. Correlation analyses were carried out using Pearson′s correlation coefficient. To identify the most predictive features, the “glmnet” package was used to apply the least absolute shrinkage and selection operator (LASSO) method. Subsequently, the “rms” package was employed to develop numeric models and conduct multivariate logistic regression analyses. LASSO regression with 10‐fold cross‐validation was used to select the optimal set of influential factors. To further confirm the variables chosen by LASSO, multiple forward stepwise logistic regression was performed. A nomogram was developed using the independent risk factors identified by multivariate logistic regression, applying the “regplot” package. Each variable′s score was added together to generate a total nomogram score, which was subsequently translated into a predicted probability. The nomogram′s discriminative performance was assessed via the concordance index (C‐index), and 500 bootstrap resamples were employed to minimize overfitting bias. A calibration curve was used to evaluate the consistency between the model′s predicted risk probabilities and the actual observed outcomes. To determine the nomogram′s clinical utility across different threshold probabilities, decision curve analysis was conducted using the “rmda” package in R. To further compare the predictive performance of our models (Model A: clinical and echocardiographic parameters with anticoagulation status; Model B: clinical and echocardiographic parameters without anticoagulation status) with the traditional CHA_2_DS_2_‐VASc model for stroke risk assessment, we plotted the ROC curves of the three prediction models in both the training and validation sets. The corresponding area under the curve (AUC) values with 95% confidence intervals (CIs) were calculated. Additionally, we calculated the net reclassification improvement (NRI) and integrated discrimination improvement (IDI) with 95% CIs to quantify the improvement in risk reclassification and discriminative ability in the validation set. Statistical significance was defined as a *p* value less than 0.05.

## 3. Results

### 3.1. Comparison of Baseline Characteristics and Multimodal Echocardiographic Parameters

According to the inclusion and exclusion criteria, a total of 402 patients with NVAF were enrolled in this study for the development and validation of a stroke risk estimation model. Of these, 289 were male (71.9%) and 113 were female (28.1%), with a mean age of 59.7 ± 10.8 years. Among the participants, 70 patients (17.4%) had experienced a stroke, whereas 332 (82.6%) had not. The patient selection process is illustrated in Figure 1.

The results of the comparisons of clinical data, biochemical indicators, and echocardiographic parameters between the stroke and nonstroke groups are shown in Tables 1 and 2. The findings of this study indicated that patients in the stroke group were significantly older and had higher CHA_2_DS_2_‐VASc scores than those in the nonstroke group (*p* < 0.05). Furthermore, the stroke group exhibited significantly lower Ccr and eGFR values compared with the nonstroke group (*p* < 0.05). The rates of persistent AF, hypertension, hyperlipidemia, and peripheral vascular disease were also markedly higher among stroke patients, with all differences being statistically significant (*p* < 0.05). In terms of anticoagulant use, the proportion of patients using these medications was significantly lower in the stroke group than in the nonstroke group (*p* < 0.05).

**Table 1:** Comparison of clinical characteristics and biochemical parameters of 402 patients with NVAF who experienced stroke.

Variable	Cohort			*p*
	Overall (*n* = 402)	No stroke (*n* = 332)	Stroke (*n* = 70)	
Demographics				
Age, years	59.69 ± 10.75	58.76 ± 11.03	64.07 ± 8.02	< 0.001
Female sex, *n* (%)	113 (28)	96 (29)	17 (24)	0.524
Body surface area, m^2^	1.75 ± 0.23	1.75 ± 0.23	1.73 ± 0.24	0.59
SBP, mmHg	128.09 ± 18.49	127.78 ± 18.68	129.59 ± 17.61	0.442
DBP, mmHg	79.92 ± 12.07	80.06 ± 11.96	79.23 ± 12.65	0.615
Medical history				
Hypertension, *n* (%)	201 (50)	150 (45)	51 (73)	< 0.001
Diabetes, *n* (%)	55 (14)	47 (14)	8 (11)	0.484
Dyslipidemia, *n* (%)	123 (31)	111 (33)	12 (17)	0.011
Vascular disease, *n* (%)	162 (40)	121 (36)	41 (59)	< 0.001
CAD, *n* (%)	77 (19)	61 (18)	16 (23)	0.484
HF, *n* (%)	25 (6)	18 (5)	7 (10)	0.171
Persistent AF, *n* (%)	185 (46)	144 (43)	41 (59)	0.029
CHA_2_DS_2_‐VASc score	2 (1, 3)	2 (1, 3)	4 (3.25, 5)	< 0.001
Laboratory data				
Scr, *μ*mol/L	85.5 ± 35.25	84.97 ± 37.43	88.03 ± 22.26	0.365
Ccr, mL/min	77.59 ± 18.47	79.15 ± 18.7	70.19 ± 15.44	< 0.001
eGFR, mL/min per 1.73 m^2^	82.69 ± 19.29	83.85 ± 19.27	77.2 ± 18.58	0.008
NT‐proBNP, pg/mL	544 (118, 1095)	505 (108, 1095)	747 (251, 1114)	0.098
Troponin I, ng/L	4 (2, 10)	4 (2, 11)	4 (3, 8)	0.712
Medication				
Anticoagulants, *n* (%)	258 (64)	238 (72)	20 (29)	0.029

*Note:* Values are mean ± SD, *n* (percentage), or median (25th, 75th percentile).

Abbreviations: DBP, diastolic blood pressure; HF, heart failure; LAVI, left atrial volume index; LV, left ventricle; LVEDV, left ventricular end‐diastolic volume; LVESV, left ventricular end‐systolic volume; SBP, systolic blood pressure.

**Table 2:** Comparison of baseline echocardiographic characteristics of 402 patients with NVAF who experienced stroke.

Parameters	Cohort			*p*
	Overall (*n* = 402)	No stroke (*n* = 332)	Stroke (*n* = 70)	
LVEDV, mL	125.58 ± 35.38	124.55 ± 33.31	130.49 ± 43.82	0.287
LVESV, mL	48.98 ± 29.27	48.44 ± 28.72	51.53 ± 31.82	0.454
LVMI, g/m^2^	118.65 ± 32.34	116.57 ± 30.78	128.55 ± 37.59	0.014
LV ejection fraction, %	63.2 ± 10.84	63.37 ± 10.64	62.37 ± 11.76	0.513
LAVI, mL/m^2^	39.91 ± 7.14	39.52 ± 7	41.74 ± 7.54	0.025
E, cm/s	89.94 ± 22.62	90.24 ± 22.56	88.5 ± 23.02	0.566
Mean E/e′ ratio	10.33 ± 3.5	9.9 ± 3.18	12.39 ± 4.16	< 0.001
LASr, %	22.91 ± 9.37	24.22 ± 9.48	16.67 ± 5.59	< 0.001
LA stiffness	0.51 ± 0.25	0.46 ± 0.23	0.72 ± 0.22	< 0.001

*Note:* Values are mean ± SD, *n* (percentage), or median (25th, 75th percentile).

Abbreviations: LASr, left atrial reservoir strain; LAVI, left atrial volume index; LV, left ventricle; LVEDV, left ventricular end‐diastolic volume; LVESV, left ventricular end‐systolic volume; LVMI, left ventricular mass index.

Comparison of echocardiographic parameters revealed no statistically significant differences between the two groups in LVEDV, LVESV, LVEF, and early mitral inflow velocity (E) (*p* > 0.05). However, the stroke group exhibited significantly higher values for LVMI, LAVI, and E/e′ ratio compared with the nonstroke group (*p* < 0.05). Furthermore, LASr was significantly reduced in the stroke group, whereas LA stiffness was markedly increased; both differences reached statistical significance (*p* < 0.05).

### 3.2. Identification of Predictive Factors

In this study, 281 patients (69.8%) were assigned to the training set and 121 patients (30.2%) to the validation set. There were no statistically significant differences in any baseline parameters between the training and validation sets (all *p* > 0.05), indicating good comparability between the two groups (see Table 3). Univariate and multivariate logistic regression analyses based on clinical data and echocardiographic measurements from the training set were used to predict the risk of stroke in patients with NVAF. The results showed that LA stiffness, LASr, hypertension, peripheral vascular disease, and use of anticoagulant drugs were independent risk factors for stroke in patients with NVAF (Table 4).

**Table 3:** Baseline characteristics of the 402 patients in the training and validation sets.

Variable	Overall (*n* = 402)	Validation set (*n* = 121)	Training set (*n* = 281)	*p*
Age, years	59.69 ± 10.75	59.6 ± 11.02	59.72 ± 10.66	0.92
Female sex, *n* (%)	113 (28)	35 (29)	78 (28)	0.906
Body surface area, m^2^	1.75 ± 0.23	1.75 ± 0.22	1.75 ± 0.24	0.861
SBP, mmHg	128.09 ± 18.49	129.09 ± 17.19	127.67 ± 19.03	0.461
DBP, mmHg	79.92 ± 12.07	80.44 ± 12.19	79.69 ± 12.03	0.572
Medical history				
Hypertension, *n* (%)	201 (50)	61 (50)	140 (50)	0.999
Diabetes, *n* (%)	55 (14)	17 (14)	38 (14)	0.999
Dyslipidemia, *n* (%)	123 (31)	30 (25)	93 (33)	0.124
Stroke, *n* (%)	70 (17)	21 (17)	49 (17)	0.999
Vascular disease, *n* (%)	162 (40)	46 (38)	116 (41)	0.616
Coronary heart disease, *n* (%)	77 (19)	22 (18)	55 (20)	0.852
History of heart failure, *n* (%)	25 (6)	4 (3)	21 (7)	0.173
Persistent AF, *n* (%)	185 (46)	61 (50)	124 (44)	0.293
CHA_2_DS_2_‐VASc score	2 (1, 3)	2 (1, 3)	2 (1, 4)	0.134
Laboratory data				
Scr, *μ*mol/L	85.5 ± 35.25	89.68 ± 51.96	83.71 ± 24.74	0.23
Ccr, mL/min	77.59 ± 18.47	78.36 ± 21.14	77.26 ± 17.23	0.616
eGFR, mL/min/1.73 m^2^	82.69 ± 19.29	81.69 ± 20.47	83.12 ± 18.79	0.511
NT‐proBNP, pg/mL	545 (118, 1095)	472 (116, 1110)	562 (121, 1095)	0.598
Troponin I, ng/L	4 (2, 10)	4 (2, 9)	5 (2, 11)	0.159
Medication				
Antiplatelet, *n* (%)	258 (64)	84 (69)	174 (62)	0.185
Echocardiographic parameters				
LVEDV, mL	125.58 ± 35.38	125.06 ± 34.54	125.81 ± 35.79	0.844
LVESV, mL	48.98 ± 29.27	48.26 ± 27.9	49.28 ± 29.88	0.743
LV mass index, g/m^2^	118.65 ± 32.34	115.78 ± 30.92	119.89 ± 32.91	0.231
LV ejection fraction, %	63.20 ± 10.84	63.34 ± 10.86	63.14 ± 10.85	0.864
LAVI, mL/m^2^	39.91 ± 7.14	39.77 ± 7.47	39.96 ± 7	0.806
E/e′	10.33 ± 3.5	10.07 ± 3.49	10.45 ± 3.5	0.323
LASr	22.91 ± 9.37	23.52 ± 9.25	22.64 ± 9.42	0.386
LA stiffness	0.51 ± 0.25	0.48 ± 0.24	0.52 ± 0.25	0.212

*Note:* Values are mean ± SD, *n* (percentage), or median (25th, 75th percentile).

Abbreviations: DBP, diastolic blood pressure; LASr, left atrial reservoir strain; LAVI, left atrial volume index; LV, left ventricle; LVEDV, left ventricular end‐diastolic volume; LVESV, left ventricular end‐systolic volume; SBP, systolic blood pressure.

**Table 4:** Univariate and multivariate logistic regression analysis of stroke in training set.

Variable	Univariate analysis		Multivariate analysis	
	OR (95% CI)	*p*	OR (95% CI)	*p*
Age, years	1.064 (1.029–1.104)	< 0.001	0.998 (0.947–1.052)	0.954
Female sex, *n* (%)	0.928 (0.449–1.823)	0.833		
Body surface area, m^2^	0.525 (0.129–1.965)	0.352		
SBP, mmHg	1.005 (0.988–1.022)	0.542		
DBP, mmHg	0.985 (0.959–1.011)	0.256		
Medical history				
Hypertension, *n* (%)	3.576 (1.797–7.658)	< 0.001	2.942 (1.198–7.764)	0.023
Diabetes, *n* (%)	0.685 (0.225–1.715)	0.457		
Dyslipidemia, *n* (%)	0.337 (0.141–0.718)	0.008	0.356 (0.117–0.955)	0.051
Vascular disease, *n* (%)	2.681 (1.434–5.121)	0.002	2.334 (1.046–5.367)	0.041
CAD, *n* (%)	0.91 (0.391–1.936)	0.815		
HF, *n* (%)	1.534 (0.482–4.152)	0.427		
Persistent AF, *n* (%)	1.708 (0.92–3.201)	0.091		
Laboratory data				
Scr, *μ*mol/L	1.007 (0.996–1.019)	0.191		
Ccr, mL/min	0.974 (0.955–0.993)	0.008	0.978 (0.947–1.008)	0.164
eGFR, mL/min per 1.73 m^2^	0.976 (0.96–0.992)	0.003	1.009 (0.982–1.039)	0.492
NT‐proBNP, ng/mL	1 (0.999–1.001)	0.471		
Troponin I, ng/L	1.001 (0.994–1.006)	0.671		
Medication				
Anticoagulants, *n* (%)	0.203 (0.101–0.388)	< 0.001	0.095 (0.037–0.222)	< 0.001
Echocardiographic parameters				
LVEDV, mL	1.001 (0.992–1.009)	0.835		
LVESV, mL	0.999 (0.988–1.009)	0.896		
LVMI, g/m^2^	1.006 (0.997–1.015)	0.153		
LV ejection fraction, %	0.999 (0.972–1.029)	0.945		
LAVI, mL/m^2^	1.027 (0.983–1.072)	0.237		
E, cm/s	0.99 (0.975–1.004)	0.15		
Mean E/e′	1.202 (1.105–1.315)	< 0.001	1.072 (0.960–1.196)	0.207
LASr, %	0.884 (0.837–0.926)	< 0.001	0.928 (0.859–0.996)	0.047
LA stiffness, per increased 0.1	1.449 (1.271–1.676)	< 0.001	1.382 (1.117–1.727)	0.003

Abbreviations: AF, Atrial fibrillation; DBP, diastolic blood pressure; HF, heart failure; LASr, left atrial reservoir strain; LAVI, left atrial volume index; LV, left ventricle; LVEDV, left ventricular end‐diastolic volume; LVESV, left ventricular end‐systolic volume; LVMI, left ventricular mass index; SBP, systolic blood pressure.

### 3.3. Development and Validation of Models

**Figure 3:**
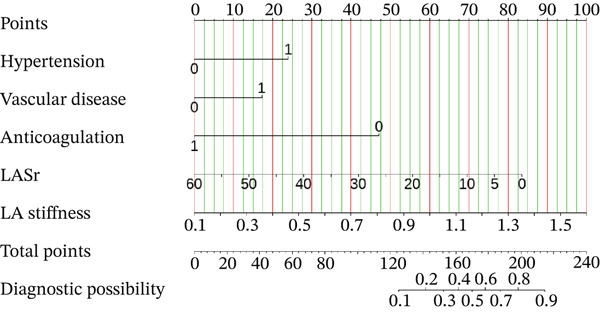
The nomogram was utilized to assess stroke risk in NVAF patients by referencing the respective probability indicated on the outcome axis.

**Figure 4:**
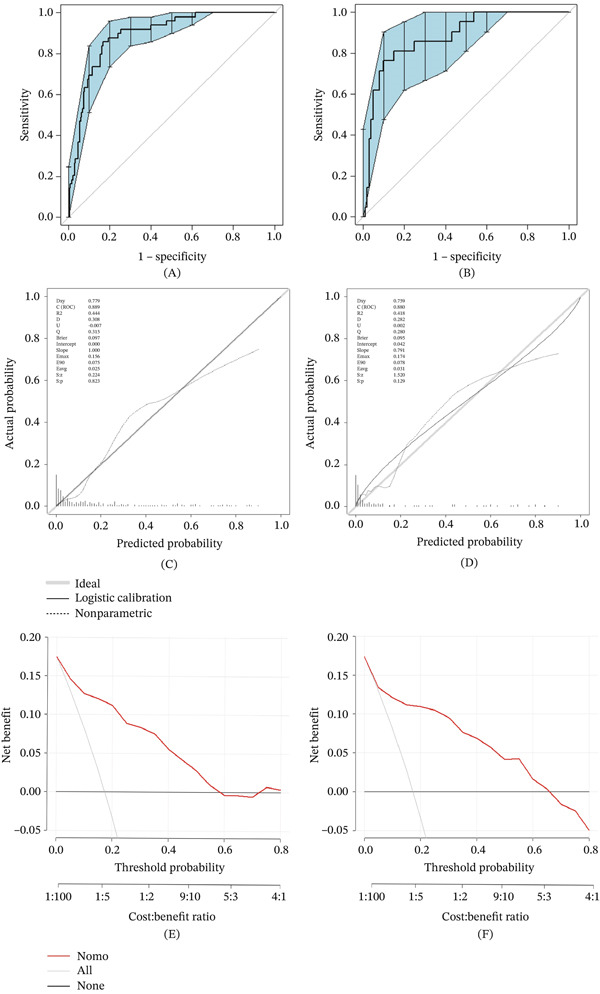
Analyses of discrimination, calibration, and decision curves in the training and validation cohorts (A, C, and E: training cohort; B, D, and F: validation cohort).

The logistic regression model was developed using the five factors listed above and integrated into the nomogram (Table 5). The nomogram was utilized to assess stroke risk in NVAF patients by referencing the respective probability indicated on the outcome axis (Figure 3). In the training set, the nomogram demonstrated good discriminative ability, with a C‐statistic of 0.889 (Figure 4A). In the validation set, the C‐index for predicting the risk of stroke was 0.880 (Figure 4B). To further assess the model′s performance, internal validation was conducted using the bootstrap method with 500 resamples. The calibration curves closely aligned with the ideal 45° diagonal, indicating good calibration accuracy for the nomogram (Figure 4C–D). In the training set, the nomogram achieved a sensitivity of 85.7% and a specificity of 83.2% for predicting stroke risk. In the validation cohort, the sensitivity and specificity were 76.2% and 89.0%, respectively. For clinical application, a nomogram score of 141.4 is recommended as the cutoff value; patients with scores above this threshold are considered to be at high risk for stroke.

**Table 5:** Multivariate logistic regression analysis in the final model coefficients.

Variables	*β*	SE	OR	95% CI	*Z*	*p*	VIF
Hypertension	1.079	0.473	2.942	1.164–7.436	2.281	< 0.023	1.055
Vascular disease	0.848	0.415	2.335	1.036–5.263	2.044	0.041	1.071
Anticoagulation	−2.353	0.455	0.095	0.039–0.232	−5.176	< 0.001	1.202
LASr, %	−0.074	0.037	0.929	0.863–0.999	−1.990	0.047	1.567
LA stiffness, per increased 0.1	0.324	0.110	1.383	1.114–1.716	0.324	0.003	1.672

Abbreviations: LA, left atrial; LASr, left atrial reservoir strain; VIF, variance inflation factor.

The clinical utility of the model was assessed using decision curve analysis. The results demonstrated that both the training and validation cohorts exhibited good net clinical benefit across a wide range of threshold probabilities. The probability range for net benefit was 0.00–0.58 in the training cohort and 0.05–0.65 in the validation cohort. As shown in Figure 4E–F, the model provided significant net benefit at various decision thresholds, indicating its effectiveness in improving stroke risk stratification in patients with NVAF.

**Figure 5:**
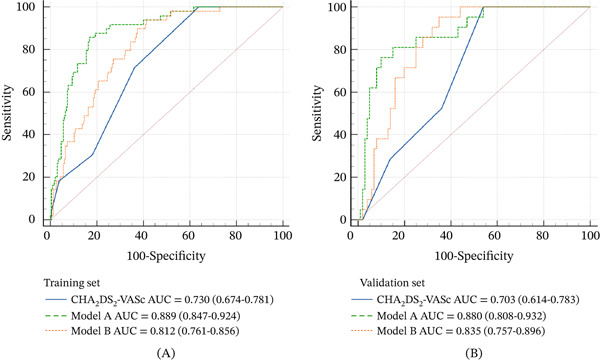
The discriminative abilities of Model A (LA stiffness, LA reservoir strain, hypertension, peripheral vascular disease, and anticoagulation), Model B (LA stiffness, LA reservoir strain, hypertension, and peripheral vascular disease), and the CHA_2_DS_2_‐VASc score for assessing stroke risk were evaluated and compared using receiver operating characteristic (ROC) curve analysis in both (A) the training set and (B) the validation set.

We also developed a predictive model based solely on clinical and echocardiographic parameters. This model (Model B: LA stiffness, LA reservoir strain, hypertension, and peripheral vascular disease) demonstrated good performance in assessing stroke risk, outperforming the traditional CHA_2_DS_2_‐VASc score, and is intended to guide clinical decision‐making for anticoagulation initiation. The ROC analysis demonstrated that Model A (LA stiffness, LA reservoir strain, hypertension, peripheral vascular disease, and anticoagulation) achieved the highest AUC values in both the training and validation sets. In the training set, the AUC for Model A was 0.889 (95% CI: 0.847–0.924), compared with 0.812 (95% CI: 0.761–0.856) for Model B (LA stiffness, LA reservoir strain, hypertension, and peripheral vascular disease) and 0.730 (95% CI: 0.674–0.781) for the CHA_2_DS_2_‐VASc model. In the validation set, Model A yielded an AUC of 0.880 (95% CI: 0.808–0.932), whereas Model B and the CHA_2_DS_2_‐VASc model achieved AUCs of 0.835 (95% CI: 0.757–0.896) and 0.703 (95% CI: 0.614–0.783), respectively. The results were shown in Figure 5.

Furthermore, we compared the performance of the three models by calculating the NRI and IDI values in the validation set (Table 6). The results indicated that Model A had an NRI of 0.531 (95% CI: 0.316–0.747, *p* < 0.01) and an IDI of 0.379 (95% CI: 0.254–0.505, *p* < 0.01) compared with the CHA_2_DS_2_‐VASc model. Model B also demonstrated significant improvement over the CHA_2_DS_2_‐VASc model, with an NRI of 0.188 (95% CI: 0.001–0.375, *p* < 0.05) and an IDI of 0.232 (95% CI: 0.142–0.321, *p* < 0.01). When directly comparing Model A with Model B, Model A showed an NRI of 0.343 (95% CI: 0.139–0.548, *p* < 0.01) and an IDI of 0.148 (95% CI: 0.077–0.219, *p* < 0.01). Hence, it is reasonable to conclude that both nomograms, particularly Model A, had superior predictive performance compared with the traditional CHA_2_DS_2_‐VASc model for stroke risk assessment in patients with NVAF.

**Table 6:** Comparisons of NRI and IDI values among the CHA2DS2‐VASc model, Model A, and Model B in the validation set.

Index	Values	95% CI	*p*
NRI			
Model A versus CHA_2_DS_2_‐VASc model	0.531	0.316–0.747	< 0.01
Model B versus CHA_2_DS_2_‐VASc model	0.188	0.001–0.375	< 0.05
Model A versus Model B	0.343	0.139–0.548	< 0.01
IDI			
Model A versus CHA_2_DS_2_‐VASc model	0.379	0.254–0.505	< 0.01
Model B versus CHA_2_DS_2_‐VASc model	0.232	0.142–0.321	< 0.01
Model A versus Model B	0.148	0.077–0.219	< 0.01

*Note:* Model A (LA stiffness, LA reservoir strain, hypertension, peripheral vascular disease, and anticoagulation); Model B (LA stiffness, LA reservoir strain, hypertension, and peripheral vascular disease).

Abbreviations: IDI, integrated discrimination improvement; NRI, net reclassification improvement.

**Figure 6:**
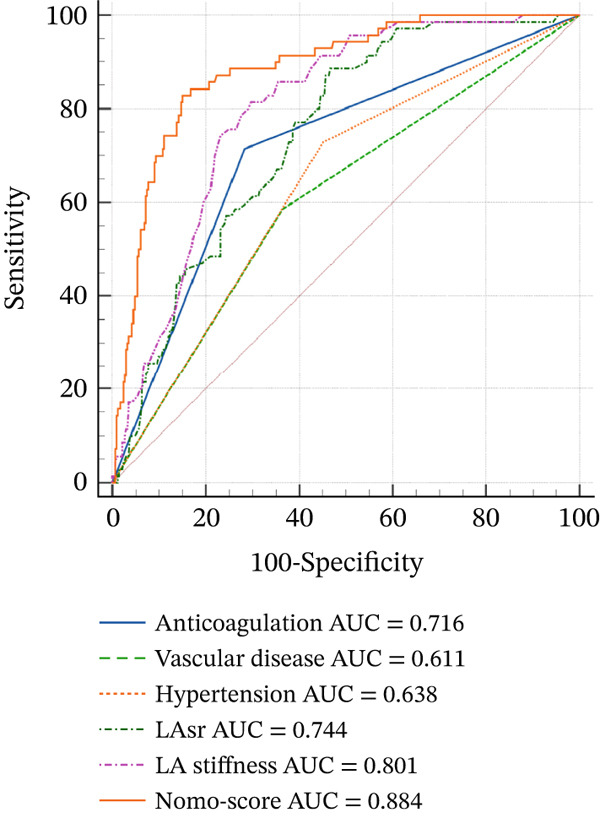
Comparative analysis of the ROC curves for predicting stroke risk in patients with nonvalvular atrial fibrillation in the entire dataset.

The sensitivity, specificity, and cutoff value of LA stiffness for predicting stroke risk were 80.4%, 70.2%, and 0.56%, respectively, and were superior to those of other transthoracic echocardiographic parameters in the entire dataset. Besides, The Nomo‐score, which incorporates LA stiffness, LASr, hypertension, peripheral vascular disease, and anticoagulant therapy, demonstrated a higher AUC (AUC = 0.884) compared with the other independent risk factors. This difference was statistically significant, as illustrated in Figure 6.

### 3.4. Correlation Between LA Stiffness and LAA Hemodynamic Parameters

**Figure 7:**
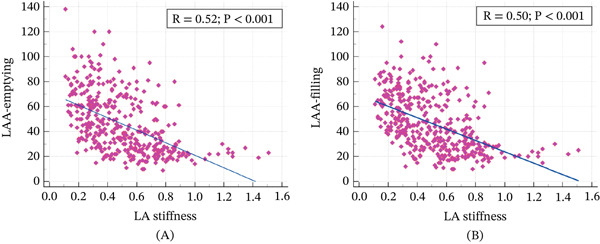
Analysis of the correlation between left atrial stiffness and left atrial appendage flow velocity. (A) The correlation analysis between left atrial stiffness and left atrial appendage emptying flow velocity. (B) The correlation analysis between left atrial stiffness and left atrial appendage filling flow velocity.

**Figure 8:**
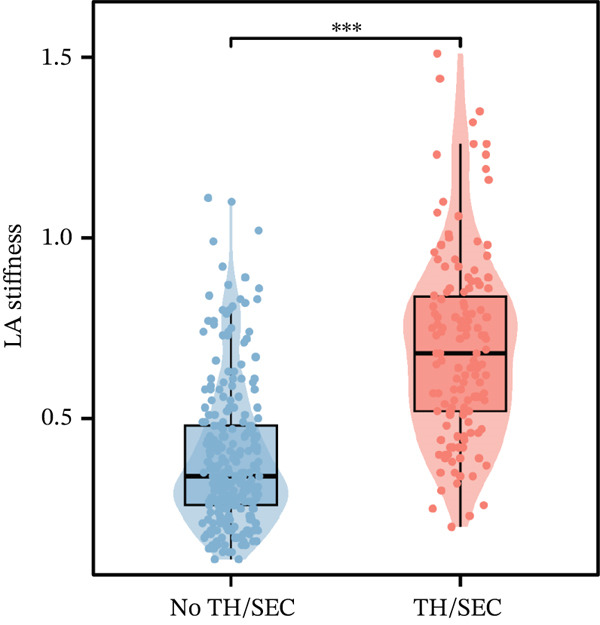
Violin plots were used to compare left atrial stiffness between the spontaneous enhancement/thrombus‐positive group and the group without these findings.

To further explore the relationship between LA stiffness and LAA hemodynamic parameters, we performed a correlation analysis. The results showed that a significant negative correlation between LA stiffness and both the emptying speed of the LAA (R = −0.52; *p* < 0.01) and the filling speed of the LAA (R = −0.50; *p* < 0.01) in all patients with NVAF (Figure 7). The violin plot indicated that LA stiffness was significantly higher in patients in the LAT/SEC (+) group compared with those in the LAT/SEC (−) group, and this difference reached statistical significance. (*p* < 0.05). The results are shown in Figure 8.

### 3.5. Reproducibility Analysis

Reproducibility analysis showed good consistency for LA stiffness measurements, with an intraobserver ICC of 0.932 (95% CI: 0.862–0.967) and an interobserver ICC of 0.927 (95% CI: 0.851–0.965), indicating excellent reliability. The results were shown in Table 7.

**Table 7:** Intraobserver and interobserver reproducibility for partial echocardiographic parameters (*n* = 30).

Characteristic	Intraobserver		Interobserver	
	ICC	(95% CI)	ICC	(95% CI)
Mean E/e′ ratio	0.904	0.799–0.952	0.897	0.650–0.960
LASr, %	0.956	0.909–0.979	0.947	0.891–0.975
LA stiffness	0.932	0.862–0.967	0.927	0.851–0.965

Abbreviations: LA, left atrial; LASr, left atrial reservoir strain.

## 4. Discussion

The results of this study indicate that: (1) LA stiffness is an independent risk factor for stroke in patients with NVAF; (2) a nomogram incorporating LA stiffness, LASr, hypertension, and peripheral vascular disease can effectively identify the risk of stroke in NVAF patients. A secondary model additionally including anticoagulation showed further improved performance but requires prospective validation; and (3) LA stiffness is significantly associated with the filling and emptying flow velocities of the LAA and patients with NVAF who also have LAT/SEC exhibit significantly higher LA stiffness compared with those without LAT/SEC. These findings may suggest that increased LA stiffness may participate in the pathogenesis of stroke in NVAF patients by affecting the hemodynamics of the LAA.

NVAF is one of the most common sustained cardiac arrhythmias and is associated with a substantial risk of ischemic stroke. In patients with NVAF, the loss of coordinated atrial contraction leads to blood stasis, particularly in the LAA, which increases the propensity for TH formation. Epidemiological studies have shown that patients with NVAF have a fivefold higher risk of stroke compared with the general population. Stroke related to NVAF tends to be particularly severe, resulting in higher rates of disability and mortality. Accordingly, timely identification and accurate risk stratification of NVAF patients at high risk of stroke are crucial for effective prevention and management strategies, including the use of anticoagulant therapy. Therefore, understanding and assessing the risk factors for stroke in NVAF patients remains a major focus of clinical research and practice.

The CHA_2_DS_2_‐VASc score is a widely used clinical tool for assessing stroke risk [12]. Building upon the original CHADS_2_ score, CHA_2_DS_2_‐VASc incorporates additional risk factors, thereby allowing for a more refined determination of stroke risk. This tool assigns points to various clinical indicators, helping physicians gain a comprehensive understanding of a patient′s stroke risk and providing a foundation for developing anticoagulation therapy strategies [13]. Previous studies have demonstrated that the CHA_2_DS_2_‐VASc score effectively identifies individuals at high risk for stroke and thromboembolic events, irrespective of prior standard anticoagulation therapy. Its use assists clinicians in pinpointing high‐risk patient groups who require tailored anticoagulation regimens. However, some studies have highlighted limitations of the CHA_2_DS_2_‐VASc score in predicting stroke risk among patients with NVAF, citing a C‐statistic value of only 0.549–0.638, which indicates relatively low sensitivity and specificity [14]. Additionally, the CHA_2_DS_2_‐VASc score cannot reliably identify patients who, despite having a low score, remain at high risk for LA TH or spontaneous echocardiographic contrast [15]. Therefore, there is a need to develop more accurate risk assessment tools to more effectively predict stroke risk in patients with NVAF. We developed a predictive model based solely on clinical and echocardiographic parameters. This model (Model B: LA stiffness, LA reservoir strain, hypertension, and peripheral vascular disease) demonstrated good performance in assessing stroke risk, outperforming the traditional CHA_2_DS_2_‐VASc score, and is intended to guide clinical decision‐making for anticoagulation initiation. We incorporated baseline anticoagulation history as a covariate to better elucidate the independent associations between echocardiographic parameters and stroke while controlling for the protective effect of anticoagulation. This model (Model A: LA stiffness, LA reservoir strain, hypertension, peripheral vascular disease, and anticoagulation) showed superior discriminative ability for stroke risk assessment. This dual‐model approach is consistent with recent literature [16], where investigators have developed risk prediction tools that account for antithrombotic treatment status to personalize risk assessment for both ischemic and bleeding events. As Claxton et al. [17] demonstrated, including anticoagulation type as a variable can help identify patients who remain at high risk for stroke despite receiving anticoagulation therapy. While CHA_2_DS_2_‐VASc and Model B remain valuable for initial decision‐making regarding anticoagulation initiation, Model A that integrates anticoagulation therapy is superior for assessing residual stroke risk in already treated NVAF patients, enabling more precise, dynamic, and patient‐centered long‐term management.

LA strain can improve the detection of patients at higher risk of stroke, especially in those with low CHA_2_DS_2_‐VASc scores [18]. The LA stiffness, defined as the ratio of early diastolic transmitral flow velocity (E) to lateral mitral annulus myocardial velocity (E/e′) divided by LASr, has emerged as a promising noninvasive marker for assessing LA hypertension and related cardiac dysfunctions [19]. This index reflects reduced LA compliance and is particularly useful in conditions such as mitral regurgitation, coronary artery disease, and heart failure with preserved ejection fraction (HFpEF). The correlation between LASI and LA hypertension is supported by several studies, which highlight its potential as an early marker for disease severity and progression [20–22]. It provides a comprehensive assessment of LA mechanics and their impact on cardiac function. LASI showed a strong correlation with LA hypertension and its ability to predict adverse outcomes in NVAF patients [23].

LA stiffness can be quantified using echocardiographic speckle‐tracking technology. When combined with LA strain measurements and clinical risk factors, this assessment can help evaluate the risk of stroke. LA stiffness is closely linked with the risk of thrombosis and stroke in NVAF [24]. Increased LA stiffness impairs atrial reservoir and contractile function, accentuating blood stasis (especially in the LAA), and thus promotes TH formation independent of traditional risk factors—even in paroxysmal or “low‐risk” NVAF settings [25]. Assessment of LA stiffness may provide critical incremental value in identifying high‐risk patients who would benefit most from preventive strategies, including anticoagulation. This study suggested that LA stiffness is significantly associated with the filling and emptying flow velocities of the LAA in NVAF patients. Besides, those patients who have LAA TH or SEC exhibit significantly higher LA stiffness compared with those without TH or SEC. These findings may suggest that increased LA stiffness may participate in the pathogenesis of stroke in NVAF patients by affecting the hemodynamics of the LAA. A previous study also found that LA stiffness significantly increased after endocardial LAA occlusion, with baseline LAA size correlating with the magnitude of stiffness increase. These findings suggest that the LAA plays a crucial role in LA mechanics, and alterations in its function or structure, such as increased stiffness, could impact stroke risk by affecting blood flow dynamics within the atrium [26].

An increase in LA stiffness is usually associated with atrial fibrosis, scar formation, or elevated LA pressure. Increased LA stiffness can result in incomplete LA emptying, leading to blood stasis in the LA [27]. This study also indicates that LA stiffness, as assessed by TEE, is higher in the group with spontaneous echocardiographic contrast and/or TH compared with the group without these findings.

The nomogram, which incorporates LA stiffness alongside traditional risk factors, is designed to improve risk stratification for stroke in patients with NVAF. Although the CHA_2_DS_2_‐VASc score remains the cornerstone of current stroke risk assessment, it does not account for structural and functional remodeling of the left atrium, which is increasingly recognized as an independent risk factor for thromboembolism. Our findings suggest that even among patients with a low CHA_2_DS_2_‐VASc score, those with significantly increased LA stiffness are at elevated risk for stroke. Therefore, the nomogram could serve as an important adjunct for clinical decision‐making, particularly in this subset of patients.

## 5. Study Limitations

This study was conducted at a single center and utilized a retrospective cohort, which may introduce selection bias and limit the generalizability of the findings to other populations or healthcare settings. Multicenter, prospective studies are needed to validate the robustness and applicability of the nomogram in more diverse populations. Although the nomogram was internally validated within the study cohort, external validation in an independent cohort was not performed. Without such external validation, the generalizability and reliability of the nomogram in other centers or ethnic groups remain undetermined. Stroke events were identified through medical records, which may carry a risk of misclassification or underestimation, particularly regarding silent or minor strokes. Recruiting patients from an AF ablation cohort introduces inherent selection bias, as these individuals represent a subgroup of NVAF patients deemed suitable for invasive intervention. Therefore, our findings may not be generalizable to the broader NVAF population. LA stiffness was measured at baseline, and any subsequent changes in atrial structure or function during follow‐up were not tracked or incorporated into the model. Dynamic changes might further influence stroke risk and the predictive value of the nomogram. In addition, assessment of LA stiffness by echocardiography can vary with loading conditions and image quality, potentially affecting the consistency and accuracy of LA stiffness measurements.

## 6. Conclusion

This study demonstrates that LA stiffness is an independent risk factor for stroke in patients with NVAF and provides incremental value to existing risk stratification tools. Furthermore, we developed and validated a nomogram for assessing the risk of stroke in NVAF patients. Notably, a secondary model that additionally incorporated anticoagulation status demonstrated superior discriminative ability, suggesting its potential value for evaluating residual stroke risk in patients already receiving anticoagulation therapy, although prospective validation is required. Meanwhile, the primary model, incorporating LA stiffness, LASr, hypertension, and peripheral vascular disease, effectively identified the association with stroke and may serve as a useful tool for initial stroke risk assessment. In addition, LA stiffness is closely associated with the filling and emptying velocities of the LAA, and patients with NVAF who present with either LAT/SEC have significantly higher LA stiffness than those without these findings. Collectively, these results suggest that increased LA stiffness may contribute to the development of stroke in NVAF patients by influencing LAA hemodynamics.

## Author Contributions

Xiangling Cao, Decai Zeng, and Ji Wu were responsible for conceptualizing and designing the study. Data collection and acquisition were carried out by Yongzhi Cai, Shuai Chang, and Tongtong Huang. Bingling Wu managed data curation. Data analysis, interpretation, and statistical procedures were performed by Xiangling Cao and Decai Zeng. The initial manuscript draft was written and revised by Xiangling Cao and Decai Zeng, with further revisions and financial support provided by Decai Zeng and Ji Wu. Decai Zeng and Xiangling Cao contributed equally to this work.

## Funding

This study was supported by Key Program of Guangxi Natural Science Foundation (2023GXNSFDA026010); Youth Science Foundation of Guangxi Medical University (10.13039/501100012543; GXMUYSF201916); and “139” Project of Guangxi Medical aimed at training high‐level backbone talents (G201903014).

## Disclosure

All authors contributed critical feedback to the final manuscript and had complete access to all data.

## Ethics Statement

This study received approval from the Ethics Committee of the First Affiliated Hospital of Guangxi Medical University (Approval Number: 2022‐KT‐077), and written informed consent was obtained from all participants involved in this study.

## Conflicts of Interest

The authors declare no conflicts of interest.

## Data Availability

The data that support the findings of this study are available on request from the corresponding author. The data are not publicly available due to privacy or ethical restrictions.
